# Establishment and validation of a predictive model for respiratory failure within 48 h following admission in patients with sepsis: a retrospective cohort study

**DOI:** 10.3389/fphys.2023.1288226

**Published:** 2023-11-09

**Authors:** Bin Wang, Jianping Chen, Maofeng Wang

**Affiliations:** ^1^ Department of Emergency, Affiliated Dongyang Hospital of Wenzhou Medical University, Dongyang, Zhejiang Province, China; ^2^ Department of Biomedical Sciences Laboratory, Affiliated Dongyang Hospital of Wenzhou Medical University, Dongyang, China

**Keywords:** sepsis, respiratory failure, predictive models, machine learning, risk factors

## Abstract

**Objective:** The objective of this study is to identify patients with sepsis who are at a high risk of respiratory failure.

**Methods:** Data of 1,738 patients with sepsis admitted to Dongyang People’s Hospital from June 2013 to May 2023 were collected, including the age at admission, blood indicators, and physiological indicators. Independent risk factors for respiratory failure during hospitalization in the modeling population were analyzed to establish a nomogram. The area under the receiver operating characteristic curve (AUC) was used to evaluate the discriminative ability, the GiViTI calibration graph was used to evaluate the calibration, and the decline curve analysis (DCA) curve was used to evaluate and predict the clinical validity. The model was compared with the Sequential Organ Failure Assessment (SOFA) score, the National Early Warning Score (NEWS) system, and the ensemble model using the validation population.

**Results:** Ten independent risk factors for respiratory failure in patients with sepsis were included in the final logistic model. The AUC values of the prediction model in the modeling population and validation population were 0.792 and 0.807, respectively, both with good fit between the predicted possibility and the observed event. The DCA curves were far away from the two extreme curves, indicating good clinical benefits. Based on the AUC values in the validation population, this model showed higher discrimination power than the SOFA score (AUC: 0.682; *p* < 0.001) and NEWS (AUC: 0.520; *p* < 0.001), and it was comparable to the ensemble model (AUC: 0.758; *p* = 0.180).

**Conclusion:** Our model had good performance in predicting the risk of respiratory failure in patients with sepsis within 48 h following admission.

## Introduction

### Background

Sepsis is the result of an invasion of pathogens into the body, leading to immune disorders and organ dysfunction (Lelubre and Vincent, 2018; Rubio et al., 2019). Due to its high mortality and disability rates, sepsis has become a global concern (Singer et al., 2016; Rhodes et al., 2017). A meta-analysis of 27 studies from seven developed countries showed that the incidence of hospital-treated sepsis was 437/100,000, and the mortality rate was 17% (Fleischmann et al., 2016). In organ dysfunction caused by sepsis, the lung is a commonly affected organ, which can easily lead to respiratory failure (Erickson et al., 2009; Cochi et al., 2016; Singer et al., 2016; Fowler et al., 2019).

Respiratory failure is a decrease in PaO_2_ caused by various reasons. In the United States, the incidence rate of respiratory failure is 1,275/100,000, and the hospital mortality rate of patients with respiratory failure is 12% (Kempker et al., 2020). It is one of the common critical diseases, and the surviving patients may have physical, psychological, and cognitive problems (Brummel et al., 2015; Hashem et al., 2016). It is considered a burden on public health and the healthcare system (Bice et al., 2013). Studies have shown that the mortality rate of sepsis patients with respiratory failure is significantly increased (Angus et al., 2001; Bellani et al., 2016; Cochi et al., 2016; Fowler et al., 2019). Therefore, it is important to predict whether respiratory failure may occur during hospitalization in patients with sepsis so that patients with a high risk can be identified early and interventions can be undertaken to reduce the mortality rate.

Studies have shown that in patients with COVID-19, the SOFA scores (Vincent et al., 1996) and NEWS (Subbe et al., 2001) are useful for predicting respiratory failure (Lalueza et al., 2022). However, the scoring systems and models mentioned previously were not developed for predicting respiratory failure, and there are doubts about their efficacy in predicting the risk of respiratory failure in patients with sepsis. Some scholars have developed a model for predicting the risk of respiratory failure in patients with pancreatitis (Qiu et al., 2022). However, as the model is not aimed at patients with sepsis, there are doubts about its efficacy in predicting the risk of respiratory failure in patients with sepsis. Several models could predict acute respiratory distress syndrome (ARDS) in sepsis patients, but they are established based on the patients from the intensive care unit, indicating limited practice with those in the ordinary ward (Bai et al., 2022; Xu et al., 2023). In addition, respiratory failure is a progressed and deteriorated status of ARDS; thus, the prediction model might not be shared by them.

The aim of our study was to develop a useful predictive model for predicting the risk of respiratory failure within 48 h following admission in sepsis patients to help clinicians screen high-risk patients for timely intervention and communication.

## Methods and materials

### Research participants

In this retrospective study, a total of 1,738 sepsis patients admitted to Dongyang People’s Hospital from June 2013 to April 2023 were included. The inclusion criteria were meeting the diagnostic criteria of sepsis 3.0; infection caused the SOFA score to increase by 2 points on the original basis. The exclusion criteria were as follows: 1) patients younger than 18 years old, 2) patients with respiratory failure at admission (patients with SpO_2_ less than 90% at admission), 3) and patients who discontinued treatment. Respiratory failure within 48 h following admission was based on the lowest PaO_2_/FiO_2_ values of less than 300 mmHg.

### Observation indicators

We collected some basic information of the patients, such as gender, age, and past medical history, of the following: hypertension, diabetes, cerebral infarction, chronic lung disease (including chronic obstructive pulmonary disease, interstitial pneumonia, and chronic pulmonary fibrosis), chronic heart disease (including cardiac insufficiency NYHA II grade and above and old myocardial infarction), chronic liver disease (cirrhosis), chronic kidney disease (chronic renal insufficiency and nephrotic syndrome), tumor, leukemia, and the involved infection sites, including the central nerve system, lung, bile duct, urinary duct, and peritonitis. We also collected the following first indicators at admission: hypersensitive C-reactive protein, procalcitonin, alanine transaminase, aspartate transaminase, triglyceride, total bilirubin, creatinine, blood urea nitrogen, lactic acid, pro-brain natriuretic peptide (Pro-BNP), cholinesterase, prothrombin time, D-dimer, activated partial thromboplastin time, potassium, sodium, magnesium, calcium, phosphorus, hemoglobin, white blood cell count, platelet count, albumin, globulin, mean arterial pressure, pulse oxygen saturation, body temperature, respiratory rate, heart rate, Glasgow Coma Score (GCS), SOFA score, and NEWS (i.e., a total of 46 indicators, two scoring systems).

### Method description

First, the data were analyzed using R software (version 4.2.2). Continuous variables with a normal distribution were expressed as the mean plus or minus standard deviation and were tested using the *t*-test. Variables with a non-normal distribution were expressed as the median and interquartile range, and were tested using the Mann–Whitney U test. Categorical variables were expressed as a percentage and were tested using the chi-squared test.

The highest missing ratio among all variables was that of lactic acid (20.3%), and the missing ratios of the other variables were all less than 20%. We set up seeds and used the mice package to perform multiple imputations. In order to facilitate clinical interpretation and to ensure that there was a linear relationship between each variable and logitp when multivariate logistic regression analysis was conducted, some continuous variables were converted to categorical variables. The data were randomly divided into the modeling population and the validation population at the ratio of 7:3. Univariate analysis was performed on the modeling population; multicollinear analysis was performed on the significant indicators in the univariate analysis, which were evaluated using variance inflation coefficients (VIFs). VIFs of less than 10 indicated that there was no notable collinearity among the risk factors (Hsieh and Lavori, 2000). Whether there was a linear relationship between the variables and logitp was tested by the Box-Tidwell function, with a *p*-value < 0.05 indicating the absence of a linear relationship. After confirming that there was no multicollinearity among the variables and that there was a linear relationship with logitp, multivariate logistic regression analysis was performed, and then, stepwise regression analysis was performed to screen out the independent risk factors and establish a nomogram.

### Evaluation of the established prediction model

The area under the receiver operation curve (AUC) was used to assess the discrimination ability of the predictive model. An AUC > 0.75 indicated that the model was good (Silva et al., 2015); a calibration graph was applied to evaluate the agreement between the predicted probability and the actual observed probability. When a *p*-value tested by Hosmer–Lemeshow was greater than 0.05, the model showed adequate fitness (Niu et al., 2017). Finally, the net clinical benefit of using the established model could be shown as the distance from the all curve to the none curve (the two extreme curves) in decision curve analysis. In another way, the more the distance of the model curve from the two extreme curves, the better the clinical significance and net benefit conferred to the clinic (Nattino et al., 2016); they were used to judge the discrimination, calibration, and clinical validity, respectively, of our model, and these were also verified in the validation population.

In addition, several other models were constructed using the NEWS and the SOFA score in the validation populations, with AUCs being compared using the DeLong test. When the significance in the DeLong test was less than 0.05, a significant difference was observed between the compared AUCs. An ensemble model based on the enrolled 46 variables was established using the ensemble methods, including support vector machines (SVMs), decision trees C5.0 (C5.0), extreme gradient boosting (XGBoost), and the decision by the following R packages: CARET, XGBoost, C50, e1071, and gbm (Jiang et al., 2022). The importance of involved variables in the ensemble method was evaluated using SHAP values.

## Results

### Comparison of the basic information between the modeling population and the validation population

**FIGURE 1:**
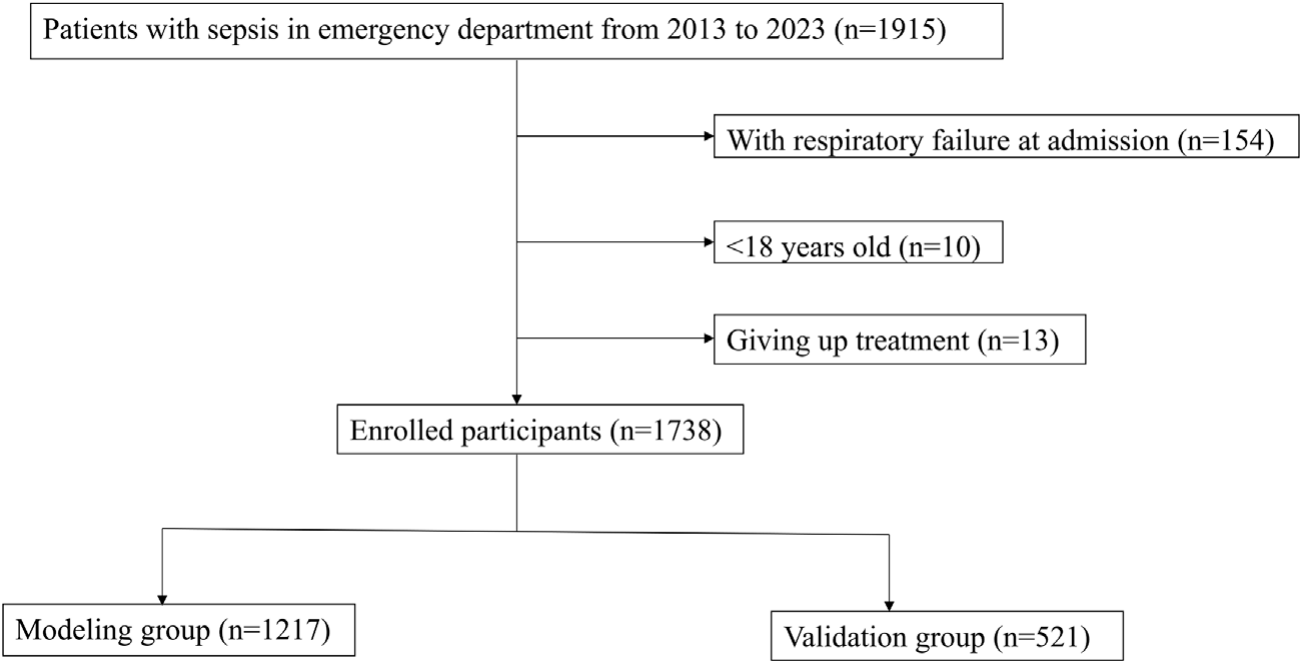
Flowchart for patient inclusion and exclusion.

A total of 1,915 patients with sepsis were included, including 154 patients with respiratory failure at admission, 13 patients who discontinued treatment, and 10 patients younger than 18 years (Figure 1). Among the study subjects, there were 345 patients with respiratory failure during hospitalization, accounting for 19.9% of the patients. The number of invasive ventilator users was 115, accounting for a percentage of 6.6%. There were 28% cases with lung infection, 10% with bile duct infection, 17% with urinary duct infection, and 45% with infection in other anatomic sites or unknown sites. A total of 1,217 patients were in the modeling population, and 521 patients were in the validation population. There was no significant difference in any variable between the modeling population and the validation population (*p* > 0.05; Table 1).

**TABLE 1:** Baseline characteristics of the modeling population and validation population^a^.

Variable	Total (*n* = 1,738)	Modeling (*n* = 1,217)	Validation (*n* = 521)	*p-*value
Gender, n (%)				0.823
Male	962 (55)	671 (55)	291 (56)	
Female	776 (45)	546 (45)	230 (44)	
Age (years)	71 (59, 81)	71 (59, 80)	70 (58, 81)	0.579
Crp (mg/L)	113.6 (47.29, 187.64)	117.1 (47.2, 187.75)	109.51 (47.57, 186.98)	0.979
Procalcitonin (ng/mL)	6.82 (1.73, 28.9)	6.93 (1.74, 29.57)	6.71 (1.62, 27.64)	0.998
Alanine transaminase (U/L)	24 (14, 44)	24 (14, 45)	24 (15, 44)	0.982
Aspartate transaminase (U/L)	33 (22, 60)	32 (22, 60)	34 (22, 58)	0.397
Triglyceride (mmol/L)	2.99 (2.4, 3.66)	3.01 (2.43, 3.66)	2.97 (2.36, 3.68)	0.791
Total bilirubin (umol/L)	12.9 (8.5, 24.37)	12.8 (8.4, 24.8)	13.2 (8.6, 23.4)	0.668
Creatinine (umol/L)	108 (75.25, 168)	109 (76, 170)	107 (74, 160)	0.306
Blood urea nitrogen (mmol/L)	8.59 (6.1, 13.28)	8.7 (6.2, 13.7)	8.24 (6, 12.4)	0.062
Lactic acid (mmol/L)	2 (1.3, 3.3)	2 (1.3, 3.4)	2 (1.3, 3)	0.367
Pro-BNP (pg/mL)	1,321 (441.28, 3,893)	1,334 (477.8, 3,821)	1,282 (404.6, 4,009)	0.392
Cholinesterase (U/L)	4,210 (3,094, 5,392)	4,219 (3,109, 5,367)	4,158 (3,032, 5,399)	0.498
Prothrombin time (s)	15.1 (14.1, 16.5)	15.1 (14.1, 16.4)	15.1 (14.2, 16.5)	0.385
D-dime (mg/L)	3.08 (1.74, 6.58)	3.13 (1.79, 6.63)	2.93 (1.61, 6.53)	0.369
Aptt (s)	42.1 (37.1, 48.1)	41.9 (36.8, 48)	42.5 (37.6, 48.4)	0.23
Potassium (mmol/L), n (%)				0.445
3.5–5.5	1,062 (61)	750 (62)	312 (60)	
<3.5	630 (36)	432 (35)	198 (38)	
>5.5	46 (3)	35 (3)	11 (2)	
Sodium (mmol/L), n (%)				0.13
135–145	876 (50)	625 (51)	251 (48)	
<135	803 (46)	546 (45)	257 (49)	
>145	59 (3)	46 (4)	13 (2)	
Magnesium (mmol/L), n (%)				0.443
0.75–1.25	1,122 (65)	793 (65)	329 (63)	
<0.75	607 (35)	419 (34)	188 (36)	
>1.25	9 (1)	5 (0)	4 (1)	
Calcium (mmol/L), n (%)				0.661
2.25–2.75	81 (5)	60 (5)	21 (4)	
<2.25	1,654 (95)	1,155 (95)	499 (96)	
>2.25	3 (0)	2 (0)	1 (0)	
Phosphorus (mmol/L), n (%)				0.15
0.97–1.61	468 (27)	322 (26)	146 (28)	
<0.97	1,168 (67)	815 (67)	353 (68)	
>1.61	102 (6)	80 (7)	22 (4)	
White blood cell (10^9^/L), n (%)				0.472
4–10	588 (34)	413 (34)	175 (34)	
<4	179 (10)	132 (11)	47 (9)	
>10	971 (56)	672 (55)	299 (57)	
Hemoglobin (10^9^/L), n (%)				0.864
110–160	981 (56)	687 (56)	294 (56)	
<110	706 (41)	496 (41)	210 (40)	
>160	51 (3)	34 (3)	17 (3)	
Platelet (10^9^/L), n (%)				0.687
100–300	1,142 (66)	795 (65)	347 (67)	
<100	497 (29)	349 (29)	148 (28)	
>300	99 (6)	73 (6)	26 (5)	
Albumin (g/L)	29.6 (26.1, 32.5)	29.6 (26.2, 32.5)	29.6 (26, 32.5)	0.733
Globulin (g/L), n (%)				0.457
20–35	1,435 (83)	1,013 (83)	422 (81)	
<20	174 (10)	115 (9)	59 (11)	
>35	129 (7)	89 (7)	40 (8)	
SpO_2_ (%)	97 (95, 98)	97 (95, 98)	97 (95, 98)	0.289
Temperature (°C), n (%)				0.361
36–37.5	746 (43)	528 (43)	218 (42)	
<36	86 (5)	65 (5)	21 (4)	
>37.5	906 (52)	624 (51)	282 (54)	
Map (mmhg), n (%)				0.612
70–105	1,091 (63)	773 (64)	318 (61)	
<70	450 (26)	308 (25)	142 (27)	
>105	197 (11)	136 (11)	61 (12)	
Heart rate (times/min)	98 (84, 114)	98 (84, 114)	100 (85, 114)	0.444
Breath rate (times/min)	20 (19, 22)	20 (18, 22)	20 (20, 21)	0.992
GCS	15 (15, 15)	15 (15, 15)	15 (15, 15)	0.577
Diabetes, n (%)				0.214
No	1,398 (80)	969 (80)	429 (82)	
Yes	340 (20)	248 (20)	92 (18)	
Hypertension, n (%)				0.952
No	1,011 (58)	709 (58)	302 (58)	
Yes	727 (42)	508 (42)	219 (42)	
Cerebral infarction, n (%)				0.611
No	1,677 (96)	1,172 (96)	505 (97)	
Yes	61 (4)	45 (4)	16 (3)	
Tumor, n (%)				0.941
No	1,441 (83)	1,008 (83)	433 (83)	
Yes	297 (17)	209 (17)	88 (17)	
Chronic lung disease, n (%)				1
No	1,676 (96)	1,174 (96)	502 (96)	
Yes	62 (4)	43 (4)	19 (4)	
Chronic heart disease, n (%)				0.971
No	1,706 (98)	1,194 (98)	512 (98)	
Yes	32 (2)	23 (2)	9 (2)	
Chronic liver disease, n (%)				0.681
No	1,639 (94)	1,150 (94)	489 (94)	
Yes	99 (6)	67 (6)	32 (6)	
Chronic kidney disease, n (%)				0.639
No	1,674 (96)	1,170 (96)	504 (97)	
Yes	64 (4)	47 (4)	17 (3)	
Leukemia, n (%)				0.233
No	1,715 (99)	1,204 (99)	511 (98)	
Yes	23 (1)	13 (1)	10 (2)	
Central nerve infection, n (%)				1
No	1,732 (100)	1,213 (100)	519 (100)	
Yes	6 (0)	4 (0)	2 (0)	
Lung infection, n (%)				0.672
No	1,258 (72)	885 (73)	373 (72)	
Yes	480 (28)	332 (27)	148 (28)	
Bile duct infection, n (%)				0.289
No	1,559 (90)	1,085 (89)	474 (91)	
Yes	179 (10)	132 (11)	47 (9)	
Urinary duct infection, n (%)				0.288
No	1,448 (83)	1,022 (84)	426 (82)	
Yes	290 (17)	195 (16)	95 (18)	
Peritonitis, n (%)				0.105
No	1,622 (93)	1,144 (94)	478 (92)	
Yes	116 (7)	73 (6)	43 (8)	
SOFA score	4 (3, 7)	4 (3, 7)	4 (3, 6)	0.626
NEWS score Ventilator, n (%)	3 (2, 5)	3 (2, 5)	3 (2, 5)	0.653
No	1,623 (93)	1,144 (94)	479 (92)	0.107
Yes	115 (7)	73 (6)	42 (8)	

Callout: a, continuous variables are described by the median and quartiles. Category varieties are analyzed by the χ^2^ test and continuous variables are analyzed by the Wilcoxon rank-sum test; b, first examination index following admission.

Crp, high-sensitivity C-reactive protein; Pro-BNP, pro-brain natriuretic peptide; Aptt, activated partial thromboplastin time; SpO_2_, pulse oxygen saturation; Map, mean arterial pressure; GCS, Glasgow Coma Score; SOFA, Sequential Organ Failure Assessment; NEWS, National Early Warning Score.

### Variable screening and logistic model construction

Univariate analysis of the modeling population showed that procalcitonin, aspartate transaminase, triglyceride, blood urea nitrogen, lactic acid, Pro-BNP, cholinesterase, prothrombin time, D-dimer, phosphorus, albumin, globulin, pulse oxygen saturation, respiratory rate, GCS, lung infection, and peritonitis were associated with respiratory failure (*p* < 0.001; Table 2). The results showed that the included variables had no collinearity in predicting respiratory failure (VIFs < 10; Supplementary Table S1), and there was a linear relationship with logitp (*p* > 0.05; Supplementary Table S2), suggesting that they could be used to construct a logistic regression model. Multivariate logistic regression analysis and stepwise regression showed that the respiratory rate, lactic acid, Pro-BNP, D-dimer, albumin, globulin, pulse oxygen saturation, GCS, lung infection, and peritonitis were independent factors related to respiratory failure (Table 3).

**TABLE 2:** Univariate analysis between respiratory failure and no respiratory failure in the modeling population^a^.

Variable	Total (*n* = 1,217)	Control group (*n* = 972)	With respiratory failure (*n* = 245)	*p*-value
Gender, n (%)				0.952
Male	671 (55)	535 (55)	136 (56)	
Female	546 (45)	437 (45)	109 (44)	
Age (years)	71 (59, 80)	71 (59, 80)	73 (58, 81)	0.364
Crp (mg/L)	117.1 (47.2, 187.75)	113.28 (45.87, 182.85)	127.81 (57.6, 200)	0.024
Procalcitonin (ng/mL)	6.93 (1.74, 29.57)	6.34 (1.63, 24.81)	12.07 (2.82, 48.38)	<0.001
Alanine transaminase (U/L)	24 (14, 45)	24 (14, 43)	26 (16, 48)	0.136
Aspartate transaminase (U/L)	32 (22, 60)	31 (21, 56)	37 (23, 80)	<0.001
Triglyceride (mmol/L)	3.01 (2.43, 3.66)	3.04 (2.51, 3.7)	2.75 (2.14, 3.46)	<0.001
Total bilirubin (umol/L)	12.8 (8.4, 24.8)	12.5 (8.3, 24.1)	14.3 (9, 27.3)	0.032
Creatinine (umol/L)	109 (76, 170)	105 (76, 167)	121 (82, 190)	0.029
Blood urea nitrogen (mmol/L)	8.7 (6.2, 13.7)	8.4 (6.04, 12.9)	10.7 (7.2, 16.73)	<0.001
Lactic acid (mmol/L)	2 (1.3, 3.4)	1.9 (1.3, 3)	3 (1.6, 4.8)	<0.001
Pro-BNP (pg/mL)	1,334 (477.8, 3,821)	1,168 (423.88, 3,032.25)	3,038 (868.9, 9,828)	<0.001
Cholinesterase (U/L)	4,219 (3,109, 5,367)	4,319.5 (3,253.5, 5,518.5)	3,730 (2,667, 4,897)	<0.001
Prothrombin time (s)	15.1 (14.1, 16.4)	15 (14.1, 16.2)	15.5 (14.2, 17.5)	<0.001
D-dime (mg/L)	3.13 (1.79, 6.63)	2.87 (1.67, 5.82)	4.66 (2.42, 10.93)	<0.001
Aptt (s)	41.9 (36.8, 48)	41.6 (36.7, 47.3)	43.9 (38, 49.6)	0.003
Potassium (mmol/L), n (%)				0.096
3.5–5.5	750 (62)	587 (60)	163 (67)	
<3.5	432 (35)	359 (37)	73 (30)	
>5.5	35 (3)	26 (3)	9 (4)	
Sodium (mmol/L), n (%)				0.005
135–145	625 (51)	503 (52)	122 (50)	
<135	546 (45)	441 (45)	105 (43)	
>145	46 (4)	28 (3)	18 (7)	
Magnesium (mmol/L), n (%)				0.043
0.75–1.25	793 (65)	648 (67)	145 (59)	
<0.75	419 (34)	321 (33)	98 (40)	
>1.25	5 (0)	3 (0)	2 (1)	
Calcium (mmol/L), n (%)				0.209
2.25–2.75	60 (5)	43 (4)	17 (7)	
<2.25	1,155 (95)	927 (95)	228 (93)	
>2.25	2 (0)	2 (0)	0 (0)	
Phosphorus (mmol/L), n (%)				<0.001
0.97–1.61	322 (26)	242 (25)	80 (33)	
<0.97	815 (67)	683 (70)	132 (54)	
>1.61	80 (7)	47 (5)	33 (13)	
White blood cell (10^9^/L), n (%)				0.151
4–10	413 (34)	332 (34)	81 (33)	
<4	132 (11)	97 (10)	35 (14)	
>10	672 (55)	543 (56)	129 (53)	
Hemoglobin (10^9^/L), n (%)				0.063
110–160	687 (56)	564 (58)	123 (50)	
<110	496 (41)	380 (39)	116 (47)	
>160	34 (3)	28 (3)	6 (2)	
Platelet (10^9^/L), n (%)				0.005
100–300	795 (65)	655 (67)	140 (57)	
<100	349 (29)	266 (27)	83 (34)	
>300	73 (6)	51 (5)	22 (9)	
Albumin (g/L)	29.6 (26.2, 32.5)	29.9 (26.78, 32.9)	27.6 (23.8, 31.3)	<0.001
Globulin (g/L), n (%)				<0.001
20–35	1,013 (83)	828 (85)	185 (76)	
<20	115 (9)	72 (7)	43 (18)	
>35	89 (7)	72 (7)	17 (7)	
SpO_2_ (%)	97 (95, 98)	97 (95, 98)	96 (93, 98)	<0.001
Temperature (°C), n (%)				0.283
36–37.5	528 (43)	422 (43)	106 (43)	
<36	65 (5)	47 (5)	18 (7)	
>37.5	624 (51)	503 (52)	121 (49)	
Map (mmhg), n (%)				0.613
70–105	773 (64)	612 (63)	161 (66)	
<70	308 (25)	252 (26)	56 (23)	
>105	136 (11)	108 (11)	28 (11)	
Heart rate (times/min)	98 (84, 114)	98 (84, 114)	105 (86, 118)	0.004
Breath rate (times/min)	20 (18, 22)	20 (18, 20)	20 (20, 23)	<0.001
GCS	15 (15, 15)	15 (15, 15)	15 (15, 15)	<0.001
Diabetes, n (%)				0.336
No	969 (80)	768 (79)	201 (82)	
Yes	248 (20)	204 (21)	44 (18)	
Hypertension, n (%)				0.119
No	709 (58)	555 (57)	154 (63)	
Yes	508 (42)	417 (43)	91 (37)	
Cerebral infarction, n (%)				0.555
No	1,172 (96)	934 (96)	238 (97)	
Yes	45 (4)	38 (4)	7 (3)	
Tumor, n (%)				0.765
No	1,008 (83)	803 (83)	205 (84)	
Yes	209 (17)	169 (17)	40 (16)	
Chronic lung disease, n (%)				0.952
No	1,174 (96)	937 (96)	237 (97)	
Yes	43 (4)	35 (4)	8 (3)	
Chronic heart disease, n (%)				0.109
No	1,194 (98)	957 (98)	237 (97)	
Yes	23 (2)	15 (2)	8 (3)	
Chronic liver disease, n (%)				0.997
No	1,150 (94)	919 (95)	231 (94)	
Yes	67 (6)	53 (5)	14 (6)	
Chronic kidney disease, n (%)				1
No	1,170 (96)	934 (96)	236 (96)	
Yes	47 (4)	38 (4)	9 (4)	
Leukemia, n (%)				1
No	1,204 (99)	961 (99)	243 (99)	
Yes	13 (1)	11 (1)	2 (1)	
Central nerve infection, n (%)				0.183
No	1,213 (100)	970 (100)	243 (99)	
Yes	4 (0)	2 (0)	2 (1)	
Lung infection, n (%)				<0.001
No	885 (73)	739 (76)	146 (60)	
Yes	332 (27)	233 (24)	99 (40)	
Bile duct infection, n (%)				0.805
No	1,085 (89)	865 (89)	220 (90)	
Yes	132 (11)	107 (11)	25 (10)	
Urinary duct infection, n (%)				0.188
No	1,022 (84)	809 (83)	213 (87)	
Yes	195 (16)	163 (17)	32 (13)	
Peritonitis, n (%)				<0.001
No	1,144 (94)	930 (96)	214 (87)	
Yes	73 (6)	42 (4)	31 (13)	
SOFA score	4 (3, 7)	4 (3, 6)	6 (4, 9)	<0.001
NEWS score	3 (2, 5)	3 (2, 4)	4 (2, 5)	<0.001

Callout: a, continuous variables are described by the median and quartiles. Category varieties are analyzed by the χ^2^ test and continuous variables are analyzed by the Wilcoxon rank-sum test; b, first examination index following admission.

Crp, high-sensitivity C-reactive protein; Pro-BNP, pro-brain natriuretic peptide; Aptt, activated partial thromboplastin time; SpO_2_, pulse oxygen saturation; Map, mean arterial pressure; GCS, Glasgow Coma Score; SOFA, Sequential Organ Failure Assessment; NEWS, National Early Warning Score.

**TABLE 3:** Multivariable logistic regression analysis and stepwise regression analysis of involved variables in the modeling group.

Variable	Multivariable logistic regression		Stepwise regression	
	OR (95% CI)	*p*-value	OR (95% CI)	*p*-value
Procalcitonin (ng/mL)	1.000 (0.995–1.004)	0.872	NA	NA
Aspartate transaminase (U/L)	0.995 (0.999–1.000)	0.072	1.000 (0.999–1.000)	0.079
Triglyceride (mmol/L)	0.970 (0.793–1.182)	0.765	NA	NA
Urea nitrogen (mmol/L)	1.007 (0.983–1.032)	0.546	NA	NA
Lactic acid (mmol/L)	1.106 (1.043–1.174)	0.001	1.108 (1.047–1.175)	<0.001
Pro-BNP (pg/mL)	1.000 (1.000–1.000)	<0.001	1.000 (1.000–1.000)	<0.001
Cholinesterase (U/L)	1.000 (0.999–1.000)	0.986	NA	NA
Prothrombin time (s)	1.002 (0.976–1.000)	0.861	NA	NA
D-dime (mg/L)	0.947 (1.021–1.081)	0.001	1.050 (1.022–1.081)	<0.001
Phosphorus <0.97 (mmol/L)	0.782 (0.538–1.144)	0.200	0.765 (0.533–1.107)	0.151
Phosphorus <1.61 (mmol/L)	1.643 (0.873–3.080)	0.122	1.741 (0.944–3.186)	0.073
Albumin (g/L)	0.947 (0.908–0.986)	0.009	0.943 (0.913–0.974)	<0.001
Globulin <20 (g/L)	2.494 (1.504–4.091)	<0.001	2.513 (1.528–4.091)	<0.001
Globulin >35 (g/L)	0.708 (0.367–1.304)	0.284	0.704 (0.366–1.290)	0.273
Breath rate (times/min)	1.047 (1.001–1.095)	0.415	1.048 (1.002–1.095)	0.039
SpO_2_ (%)	0.854 (0.805–0.905)	<0.001	0.856 (0.807–0.907)	<0.001
GCS	0.782 (0.625–0.981)	0.031	0.776 (0.624–0.967)	0.022
Lung infection	2.070 (1.470–2.914)	<0.001	2.060 (1.466–2.892)	<0.001
Peritonitis	2.788 (1.566–4.909)	<0.001	2.809 (1.590–4.908)	<0.001

Callout: Pro-BNP, pro-brain natriuretic peptide; SpO_2_, pulse oxygen saturation; GCS, Glasgow Coma Score.

### Establishment of a nomogram in the modeling population

**FIGURE 2:**
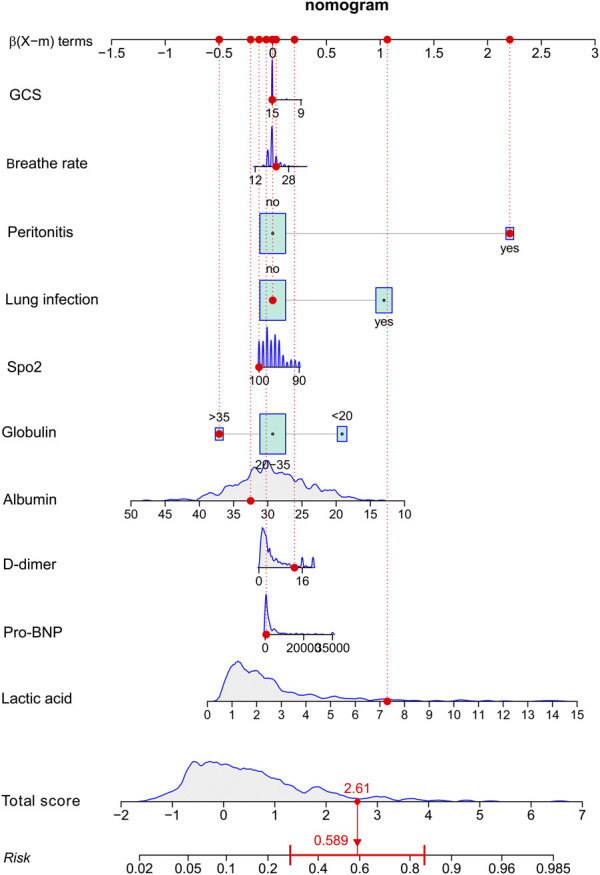
Risk-prediction nomogram for respiratory failure within 48 h following admission in patients with sepsis.

A nomogram was established using 10 variables (Figure 2). Each variable of the patient could be matched with the point scoring line according to the specific values to obtain the respective scores. Finally, the scores of each variable were summed to obtain the total score, and then, the corresponding risk could be obtained with the risk scale at the bottom.

### Model evaluation of the discrimination power, goodness of fit, and clinical benefit

**FIGURE 3:**
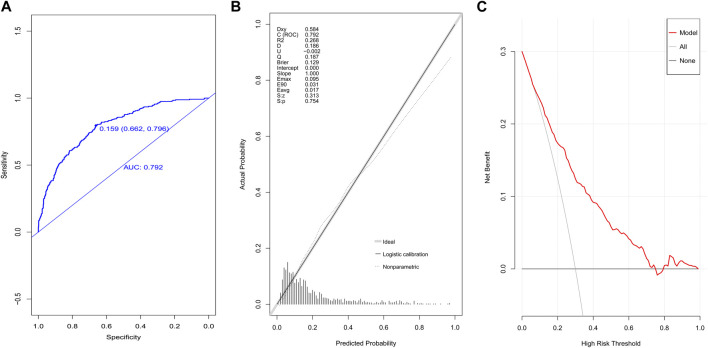
Evaluation of the prediction model in the modeling group. **(A)** ROC curves, **(B)** calibration chart, and **(C)** DCA curves.

The AUC value of the model in the modeling population was 0.792, and the 95% CI was 0.761–0.823, suggesting good discrimination (Figure 3A). The *p*-value of the calibration graph was 0.754, indicating a good fit (Figure 3B). In the DCA curve diagram, the curve of the model was far away from the two extreme curves, showing that the model had a good clinical benefit value (Figure 3C).

**FIGURE 4:**
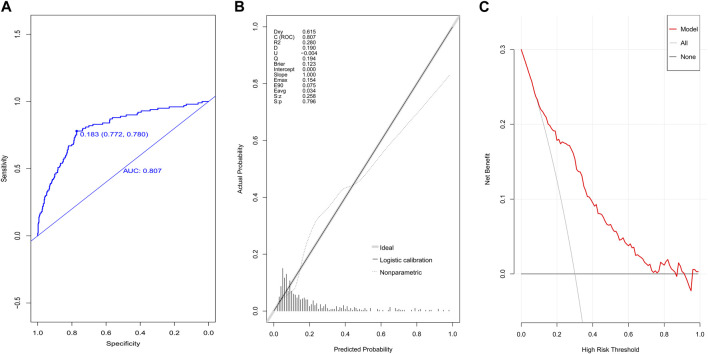
Evaluation of the prediction model in the validation group. **(A)** ROC curves, **(B)** calibration chart, and **(C)** DCA curves.

The AUC value in the validation population was 0.807, the 95% CI was 0.759–0.856 (Figure 4A), and the *p*-value of the calibration graph was 0.796, suggesting a good fit (Figure 4B). In addition, the DCA curve was far away from the two extreme curves (Figure 4C).

### Comparison with the models established by the SOFA score, NEWS, and ensemble method

**FIGURE 5:**
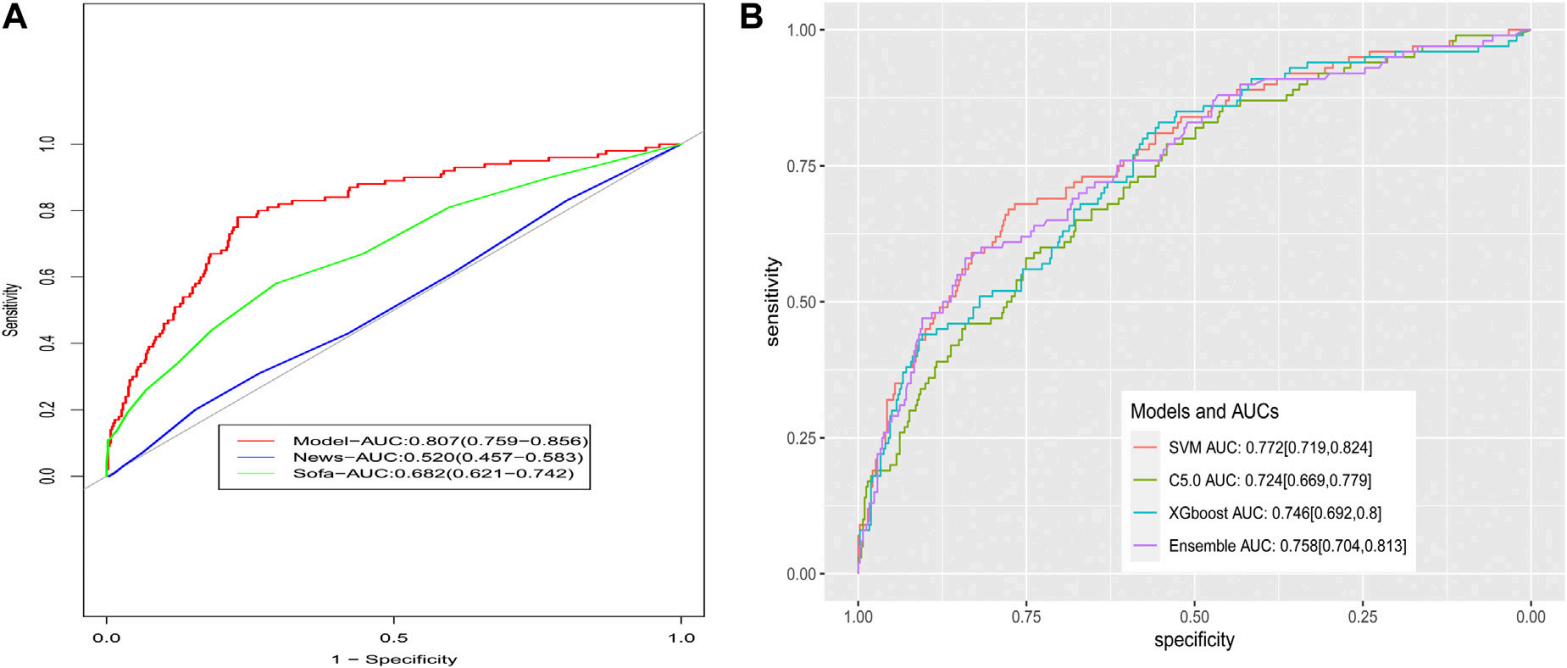
Comparison of ROCs for models. **(A)** Comparison to the models based on the SOFA and NEWS scoring system and **(B)** comparison to the models based on the ensemble method.

In the validation population, the AUC value of the model established by the NEWS was 0.520 (95% CI: 0.457–0.583). The AUC value of the model established by the SOFA score was 0.682 (95% CI: 0.621–0.742; Figure 5A). Our model had better discrimination than the NEWS model (*p* < 0.001) and the SOFA score model (*p* < 0.001). The ensemble model in our study had an AUC value of 0.758 in the validation population, with a 95% CI of 0.704–0.813 (Figure 5B), which was comparable to the established logistic prediction model (*p* = 0.180). The calibration curve indicated that the ensemble model was poorly calibrated (Supplementary Figure S1). Pro-BNP, lactic acid, SpO_2_, albumin, and creatinine were the top five variables among the 29 important variables in the ensemble model, as indicated by SHAP values (Supplementary Figure S2).

## Discussion

In this retrospective study, 10 variables were identified as independent risk factors for respiratory failure after hospital admission in septic patients, namely, respiratory rate, lactic acid, Pro-BNP, D-dimer, albumin, globulin, pulse oxygen saturation, GCS, lung infection, and peritonitis. After evaluating the discrimination, calibration, and clinical validity, our model has a good potential clinical value and interpretability. Because the prediction model incorporates 10 indicators that can be obtained as soon as patients are admitted to the hospital, it could be a helpful tool in screening for sepsis patients with a high risk of respiratory failure within 48 h following admission.

In our model, D-dimer acted as a biomarker of abnormal hemostasis and an indicator of intravascular thrombus formation (Johnson et al., 2019). The elevated D-dimer level indicates the presence of thrombus in the microvessels or large blood vessels of the lung, which can lead to imbalance of the ventilatory blood flow. Recent studies have pointed out that in patients with COVID-19, high levels of D-dimer are significantly related to respiratory failure (Takeshita et al., 2021). Some studies have pointed out that the albumin level at admission can predict the risk of respiratory failure (Thongprayoon et al., 2020), which may be related to the fact that low albumin indicates malnutrition, which leads to the atrophy of the diaphragm and respiratory muscles and affects the respiratory function. Low albumin also leads to a decrease in the plasma osmotic pressure, which is more likely to cause pulmonary edema (Blunt et al., 1998). Globulin is composed of immunoglobulin and complements, playing a key role in the antivirus and anti-bacterium response. In this study, a lower globulin level was associated with increased respiratory failure risk, which could be indirectly explained by the fact that globulin is associated with sepsis severity and lung injury would more likely occur in patients with severe sepsis (Nenke et al., 2015; Horvath-Szalai et al., 2018). Patients with a low GCS have reduced airway protection ability, which can easily lead to sputum blockage and aspiration. In some studies, GCSs have also been found to be associated with respiratory failure (Lu et al., 2022). Inflammation in the lungs would cause dysfunction in mucosal immunity, macrophages, and monocytes, which might consequently increase the susceptivity to secondary infections and respiratory distress (Bouras et al., 2018; Roquilly et al., 2020). SpO_2_ is a sensitive indicator representing whether the patient is hypoxic, and the increase in the respiratory rate is often due to the compensation caused by hypoxia. In patients with sepsis, lung damage is prone to occur (Tasaka et al., 2008), and the indicators can be reflected in the decrease of SpO_2_ and the increase of the respiratory frequency at admission; thus, these patients may be more prone to respiratory failure. Lactic acid is an indicator of the oxygen metabolism and is used to gauge the prognosis in sepsis and other critical diseases (Liu et al., 2015; Houwink et al., 2016; Luo and He, 2023), and it could serve as a predictive factor for respiratory failure in this study and ARDS in previous data (Mikkelsen et al., 2013). Pro-BNP, as an indicator of cardiac function, could also be an indicator for other disease deterioration, and its elevated levels would be closely related to respiratory failure (Takasu et al., 2013; Cheng et al., 2015). A high respiratory rate at admission indicated the presence of hypoxia and the need for compensation, which would cause fatigue in the respiratory muscle. Moreover, the presence of peritonitis was the risk factor for respiratory failure, which might be caused by surgical operation for the diagnosis and therapy of peritonitis.

The NEWS is helpful in identifying patients with early critical illnesses (Subbe et al., 2001), and the SOFA score is clinically used to predict the severity of sepsis patients (Vincent et al., 1996). Although these two scoring systems were not developed for the prediction of respiratory failure, previous studies have shown that in some patients, the aforementioned two scoring systems are helpful in predicting the risk of respiratory failure (Lalueza et al., 2022). According to the results of the current study, the AUC value of our model was significantly higher than those of the models using the aforementioned two scoring systems, suggesting higher discrimination of our model. The increased prediction power of our model could be explained by the inclusion of the variables related to the worsening of the gas exchange function (such as respiratory rate and lactic acid); however, some of them are expensive and not routinely tested in clinical practice. Our findings raise the significance of novel variables in predicting the respiratory failure risk.

We also built an ensemble model, which was comparable to our logistic model on discrimination, but lower fitness was found for the ensemble model. Moreover, the decision support provided by models based on machine learning algorithms is often difficult to be explained clinically (Kelly et al., 2019) due to its complexity. Thus, we believe that our logistic model is more functional in prediction in terms of clinical interpretation than machine learning models, such as the ensemble model.

Our study has the following limitations: It is a single-center study without validation with external data, so it is unclear if it is also applicable in other regions. There were 10 variables enrolled in the final model; some of them might be not available for a source-limited region. Our study could serve as a reference, especially our flowchart on variable selection and model evaluation, for other research workers who would like to predict the possibility of respiratory failure.

## Conclusion

Based on clinical indicators, we constructed a model for predicting the risk of respiratory failure within 48 h following admission in sepsis patients. This established model is effective, and it can help clinical staff in the decision-making process involved in the management of patients with sepsis.

## Data Availability

The raw data supporting the conclusion of this article will be made available by the authors, without undue reservation.
