# Supplementary material for: Establishment and validation of a predictive model for respiratory failure within 48 h following admission in patients with sepsis: a retrospective cohort study
Source: Front Physiol. 2023 Nov 9;14:1288226. doi: 10.3389/fphys.2023.1288226 (PMC10665857; doi:10.3389/fphys.2023.1288226)
Supplement: Supplementary file 1 [file DataSheet1.pdf]

**Supplementary table 1. Vif of variables in modeling group**

| Variables | VIF |
|---|---|
| Cholinesterase | 2.038 |
| Albumin | 1.808 |
| Blood urea nitrogen | 1.466 |
| Triglyceride | 1.374 |
| Phosphorus>1.61 | 1.312 |
| Phosphorus<0.97 | 1.309 |
| Lactic acid | 1.276 |
| Procalcitonin | 1.217 |
| Prothrombin time | 1.207 |
| Pro-BNP | 1.201 |
| Aspartate transaminase | 1.155 |
| D-dime | 1.129 |
| GCS | 1.086 |
| Breathe rate | 1.086 |
| SpO2 | 1.076 |
| Globulin>35 | 1.066 |
| Globulin<20 | 1.063 |
| Lung infection | 1.063 |
| Peritonitis | 1.054 |

Callout: Pro-BNP, pro-brain natriuretic peptide; SpO2, pulse oxygen saturation; GCS, Glasgow coma score.

**Supplementary table 2: the P value between variables and logitp**

| Variables | Pr(>\|z\|) |
|---|---|
| Procalcitonin | 0.759 |
| Aspartate transaminase | 0.694 |
| Triglyceride | 0.629 |
| Blood urea nitrogen | 0.263 |
| Lactic acid | 0.218 |
| Pro-BNP | 0.278 |
| Cholinesterase | 0.741 |
| Prothrombin time | 0.459 |
| D-dime | 0.733 |
| Albumin | 0.946 |
| Breathe rate | 0.789 |
| SpO2 | 0.426 |

Callout: Pro-BNP, pro-brain natriuretic peptide; SpO2, pulse oxygen saturation.

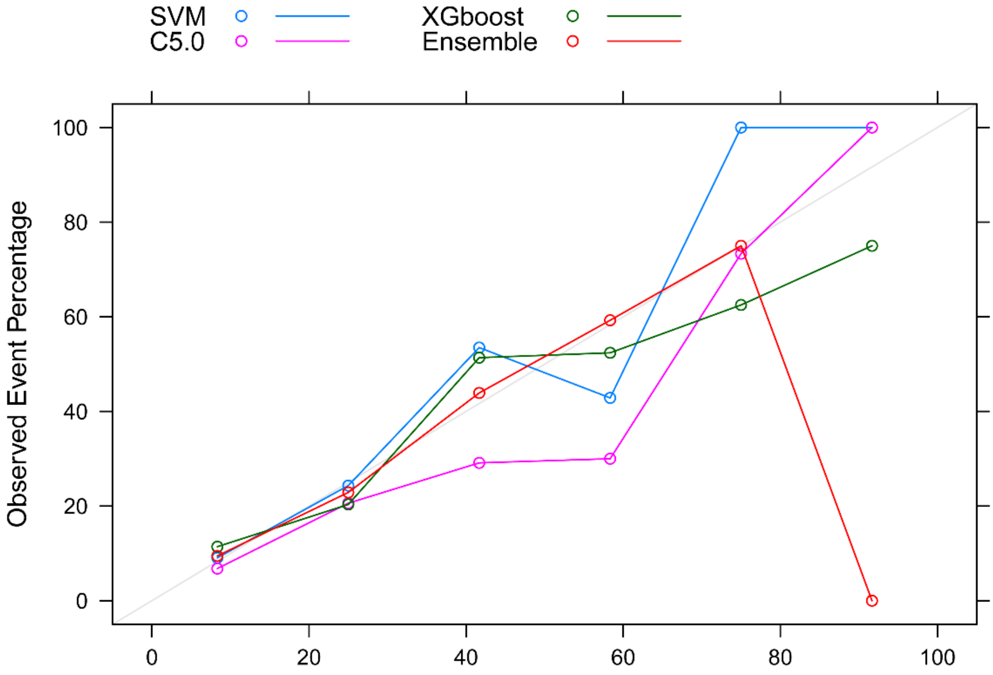


**Supplementary Fig.1. Calibration curve of ensemble model.**

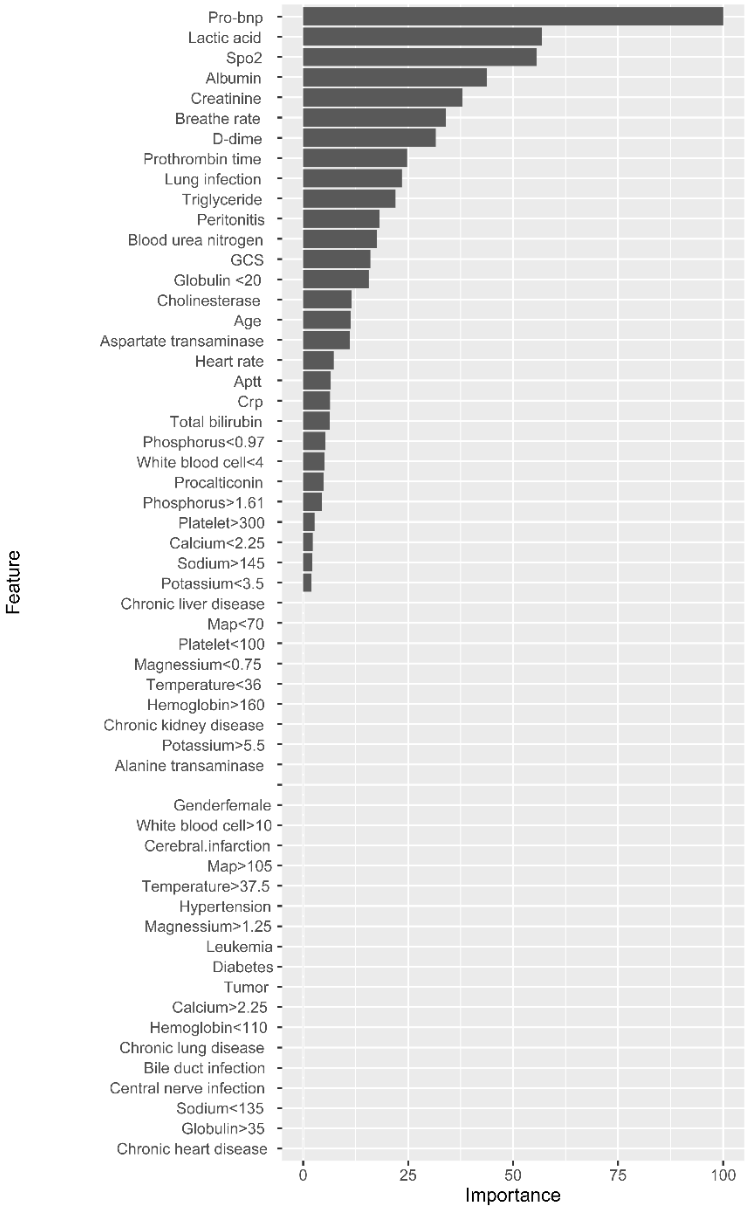


**Supplementary Fig.2. The important variables in ensemble model.**
